# Protective role of astaxanthin against bisphenol A induced biochemical and histopathological alterations in rat kidneys

**DOI:** 10.22038/ijbms.2025.88356.19081

**Published:** 2025

**Authors:** Burcu Gültekin, Seda Çetinkaya Karabekir, İlknur Çınar Ayan, Hasan Basri Savaş, Serpil Kalkan

**Affiliations:** 1 Necmettin Erbakan University, Faculty of Medicine, Department of Histology and Embryology, Konya, Türkiye; 2 Bakırçay University, Faculty of Medicine, Department of Histology and Embryology, Izmir, Türkiye; 3 Necmettin Erbakan University, Faculty of Medicine, Department of Medical Biology, Konya, Türkiye; 4 Mardin Artuklu University, Faculty of Medicine, Department of Medical Biochemistry, Mardin, Türkiye

**Keywords:** Apoptosis, Astaxanthin, Bisphenol A, Kidney diseases, Oxidative stress

## Abstract

**Objective(s):**

This study investigates the ability of astaxanthin (ASTX), a powerful anti-oxidant, to protect kidney tissue from oxidative and cellular damage resulting from bisphenol A (BPA) toxicity, a widespread global toxin associated with chronic kidney disease.

**Materials and Methods:**

We used 32 male Wistar Albino rats, 16 weeks old, and weighing 250–300 g. The rats were randomly divided into four groups: Control, Sham, BPA, and BPA+ASTX. Following the experiment, serum samples were assessed for Paraoxonase 1 (PON1), Arylesterase (ARE), urea, and creatinine levels. Changes in kidney tissue induced by BPA were examined using histopathological methods. Also, the levels of apoptosis and collagen content were evaluated.

**Results:**

ASTX treatment reversed the BPA-induced inhibition of PON1 and ARE levels, restoring them to control levels, and reduced the BPA-induced increase in urea levels. Creatinine levels showed no significant differences across the groups. BPA exposure in kidney tissue caused vacuolization, congestion, tubular dilatation, desquamation, infiltration, and increased collagen around glomeruli and blood vessels. However, ASTX treatment significantly improved these pathological findings. While BPA induced apoptosis as indicated by Bax and Bcl-2 analysis, ASTX treatment partially inhibited this process.

**Conclusion:**

These findings indicate that ASTX may protect against BPA-induced renal injury. However, the study’s limitations include the use of a single dose and a focus solely on kidney tissue. Additionally, the lack of dose-response data and evaluations of other organs or long-term effects are significant drawbacks. Future research should explore multiple doses and longer observation periods for a better understanding of ASTX’s protective efficacy.

## Introduction

Pollution involving hazardous chemicals and metals may stem from natural processes or human activities, with many of these pollutants inducing oxidative stress. For decades, oxidative stress has been implicated in the pathogenesis of numerous human diseases. Reactive oxygen species (ROS) are cytotoxic molecules capable of damaging nucleic acid bases, lipids, and proteins, ultimately resulting in cell death (1-3). Bisphenol A (BPA), along with octylphenols and nonylphenols, is an industrial pollutant classified as an endocrine-disrupting compound (EDC). BPA is commonly present in consumer plastic products, food packaging, resin-based materials, and some dental applications (4). As a result, human exposure to BPA is frequently unavoidable.

The kidney is a primary target organ for the accumulation of EDCs. In individuals with kidney disease, reduced kidney function and proteinuria are frequently observed, both of which adversely impact the recovery process (5). A study conducted in China demonstrated that reduced kidney function is associated with decreased urinary excretion of BPA. A correlation between urinary BPA excretion and albuminuria was demonstrated, revealing a potential link between BPA exposure and kidney damage (6). Renal dysfunction induced by BPA exposure impairs the elimination of BPA via the kidneys, thereby creating a vicious cycle that amplifies the toxic effects of continued BPA accumulation (7, 8). Histopathological assessments of rats exposed to BPA revealed infiltrative and dilatative alterations in kidney tissues, ultimately leading to renal failure (9). Furthermore, BPA is known to directly impact the mitochondria of kidney cells, inducing oxidative stress and mitochondrial dysfunction, ultimately causing damage to the entire organ (10). Indeed, exposure to BPA is a major source of oxidative damage in the body originating from the electron transport chain (ETC) located in the inner mitochondrial membrane (11). Reactive oxygen species (ROS) may be generated within the ETC, particularly during electron transfer from NADH to ubiquinone via the flavin mononucleotide and ubiquinone-reducing sites of complex I. Additionally, increased ROS production due to electron leakage has been reported at complex III. As a result, the increased levels of ROS impair mitochondrial energy metabolism and lead to tissue dysfunction (12).

Under pathophysiological conditions, endogenous antioxidant defenses become inadequate, necessitating external antioxidant supplementation. This helps neutralize ROS and prevents their detrimental effects on organs (13). Astaxanthin (3,3’-dihydroxy-b,b’-carotene-4,4’-dione, ASTX) is a natural carotenoid known for its potent antioxidant properties, which are attributed to its ability to neutralize free radicals. It is commonly found in marine organisms, including algae, crustaceans, salmon, shrimp, and crabs (14). Unlike other carotenoids, ASTX contains oxygenated groups in each of its ring structures, which imparts high polarity and substantially enhances its protective role against degenerative conditions. This property plays a key role in making ASTX a more potent antioxidant than β-carotene. Other studies comparing ASTX to antioxidant molecules like α-tocopherol and vitamin E have demonstrated that ASTX is more effective (15). Oxidative damage to mitochondria results in reduced oxidative phosphorylation efficiency, leading to increased mitochondrial permeability and the release of pro-apoptotic factors that initiate apoptosis (16). Research has demonstrated the protective effects of ASTX against oxidative damage in the liver, brain, and cardiovascular system (17). In addition, its therapeutic effects have been confirmed in *in vitro* epidermal-dermal interaction models of skin damage, primarily due to its anti-inflammatory properties (18). Moreover, ASTX has demonstrated a protective effect in an experimental model of ovarian damage induced by 3-nitropropionic acid (3-NPA) in rats (19). In another study, ASTX was found to exert protective effects against torsion/detorsion-induced injury through the modulation of autophagy (20). ASTX also demonstrates activity in anti-inflammatory and anti-apoptotic pathways, inhibiting mitochondrial dysfunction induced by oxidative stress, and providing protective effects to cells and organs in various pathological conditions (21). Studies have shown that ASTX exerts its antioxidant effects by increasing the activities of antioxidant enzymes such as SOD, GSH-Px, and CAT, while its anti-inflammatory effects are mediated through the modulation of the nuclear factor kappa-B (NF-κB) signaling pathway (22). Given the reasons above, the protective effects of ASTX on kidney cells may stem from its direct or indirect impact on mitochondria.

Paraoxonase-1 (PON1) and Arylesterase (ARE) are enzymes in the esterase group, encoded by the same gene, and are known to exhibit antioxidant properties (23). In the present study, oxidative damage induced by BPA exposure was evaluated by determining the levels of PON1 and ARE molecules.

This study aimed to investigate the biochemical changes in the blood serum and the histopathological alterations in the kidney tissue of rats exposed to BPA, as well as to reveal the therapeutic effects of ASTX on these changes.

## Materials and Methods

### Animals and experimental groups

Thirty-two male Wistar Albino rats (16 weeks old, weighing 200–250 g) were used in this study. The experimental protocol was conducted in accordance with the Declaration of Helsinki and was approved by the Local Ethics Committee of XX University on December 30, 2022 (Approval No. 2021-071). All animals were housed under standard laboratory conditions (temperature: 24 ± 1 °C, humidity: 45 ± 5%, light/dark cycle: 12 hr) and were fed standard laboratory chow and tap water *ad libitum*. The rats were randomly divided into four groups, with eight rats in each:

Group 1 – Control: No treatment was administered.

Group 2 – Sham (Olive Oil): 20 mg/kg of olive oil was administered via gavage once daily for two weeks.

Group 3 – BPA: 250 mg/kg of BPA, dissolved in olive oil, was administered via gavage once daily for two weeks (24).

Group 4** –** BPA + ASTX (20 mg/kg): 250 mg/kg BPA, dissolved in olive oil, was administered via gavage daily for two weeks. Two hours after each BPA administration, 20 mg/kg ASTX, also dissolved in olive oil, was administered via gavage (25).

In the study by Mashay Al-Anazi *et al*. (2022), BPA doses of 125, 250, and 500 mg/kg were administered based on previously reported oral LD50 values. They reported toxic effects at both 250 and 500 mg/kg doses. In our study, we selected the 250 mg/kg dose of BPA, as it demonstrated toxic effects while still being considered within a range that could be defined as safe (26). In the study by Toktay *et al*. (2023), ASTX was administered at doses of 10, 20, and 40 mg/kg, and it was found that the 20 mg/kg dose significantly inhibited markers of oxidative damage. Although they also reported that the 40 mg/kg dose achieved similar inhibition, the duration of administration was limited to 7 days. In our study, based on these literature findings, we selected the 20 mg/kg dose as a more reliable option and administered it for 2 weeks (27). Both BPA (≥99%, CAS No. 80-05-7, Sigma-Aldrich) and ASTX (pure 98%, CAS No. 472-61-7, Sigma-Aldrich) were freshly prepared before each administration. At the end of the 14 days, blood samples and kidney tissues were collected under anesthesia induced by intraperitoneal injection of 50 mg/kg Ketamine HCl and 10 mg/kg Xylazine HCl.

### Biochemical analyses

All collected samples were stored under appropriate conditions until the day of the experiment. Paraoxonase 1 (PON1) and Arylesterase (ARE) were analyzed as oxidative stress parameters (28). To evaluate kidney function, urea and creatinine levels were measured in serum tissue (29). PON1 (Rel Assay Diagnostics Kits, Cat. no: RL0031, Mega Tıp San., Gaziantep, Turkey), ARE (Rel Assay Diagnostics Kits, Cat. no: RL0055, Mega Tıp San., Gaziantep, Turkey), Urea (Urea Assay Kit, Cat. no: OttoBC157, Otto Scientific, Ankara, Turkey), and Creatinine (Creatinine Assay Kit, Cat. no: OttoBC139, Otto Scientific, Ankara, Turkey) levels were determined according to the manufacturer’s procedures.

### Histological examination

Following blood sample collection, the rats were euthanized, and the abdominal cavities were opened for macroscopic examination of the kidneys. After macroscopic evaluation, the excised kidney tissues were immersed in 10% neutral buffered formalin for 48 hr for fixation. Subsequently, tissues underwent routine histological processing, after which they were embedded in paraffin. Sections of 4 μm thickness were obtained from each paraffin block using a microtome (Leica RM2125RTS).

### Hematoxylin and eosin (H&E) staining

Histological analysis of kidney tissue sections stained with hematoxylin and eosin (H&E) was performed based on the evaluation of several parameters, including congestion, vacuolization, tubular dilatation, desquamation, and mononuclear cell infiltration. Histopathological scoring was conducted according to the severity of the observed alterations, categorized as follows: no change [0], mild change [1], moderate change [2], and severe change [3]. Scores for each of the five parameters, each ranging from 0 to 3, were summed to yield a total histological score ranging from 0 to 15 (30). All histopathological evaluations were performed in a double-blinded manner using a Zeiss Lab.A1 light microscope and a Zeiss AxioCam ERc 5s camera imaging system.

### Masson’s trichrome staining

Masson’s trichrome staining was performed on kidney tissue sections to evaluate the extent of fibrosis. In the stained sections, the amount of collagen fibers surrounding the Bowman’s capsule, blood vessels, and the basal lamina of renal tubules was visualized and assessed under a light microscope (31).

### Immunohistochemistry (IHC) analysis

İn the immunohistochemical analysis, primary antibodies against Bax (Santa Cruz Biotechnology, catalog no: sc-23959) and Bcl-2 (Santa Cruz Biotechnology, catalog no: sc-7382) were employed. Four-micrometer-thick paraffin-embedded tissue sections were deparaffinized by immersion in xylene for 30 min. Following deparaffinization, sections were incubated in Super Block solution (ScyTek Laboratories, Logan, UT, USA) for 10 min, then rinsed with phosphate-buffered saline (PBS) for five minutes. Primary antibody incubation was performed overnight at 4 °C. After washing with PBS for five minutes, sections were incubated with the secondary antibody for 20 min, followed by another 5-min PBS wash. Subsequently, streptavidin-peroxidase was applied for 20 min and washed again with PBS. The chromogenic substrate AEC (Sigma Aldrich, AEC101-1KT) was then added and incubated for 15 min, followed by rinsing with distilled water for five minutes. Mayer’s hematoxylin was used as a counterstain once the immunoreactivity became microscopically evident. Slides were mounted using an aqueous mounting medium and examined under a Zeiss Lab.A1 light microscope, with images captured using the Zeiss AxioCam ERc 5s camera system. Immunoreactivity was semi-quantitatively scored based on staining intensity as follows: no staining [0], weak staining [1], moderate staining [2], and strong staining [3] (32). All histopathological evaluations were performed independently by two investigators blinded to the experimental groups.

### RT-qPCR analysis

For molecular analyses, total RNA was isolated from one-third of the kidney tissue obtained from each experimental group, previously stored at –80 °C. RNA extraction was performed using RiboEX Total RNA Isolation Reagent (GeneAll, 301-001). To eliminate potential genomic DNA contamination, each sample was treated with DNase I enzyme (Thermo Fisher Scientific, #EN0521). Subsequently, cDNA was synthesized using the iScript™ cDNA Synthesis Kit (Bio-Rad, 170-8891), following the manufacturer’s instructions.

Primers for the target genes (Bax, Bcl-2, Col1A1, Col3A1) and the housekeeping gene (GAPDH) were designed using the IDT PrimerQuest Tool (https://www.idtdna.com/PrimerQuest/Home/Index). mRNA expression levels were quantified using 5x HOT FIREPol® EvaGreen® qPCR Master Mix Plus (ROX) (Solis BioDyne). Reactions were performed on a Bio-Rad CFX Connect real-time PCR system with the following thermal cycling conditions: initial activation at 95 °C for 12 min, followed by 40 cycles of denaturation at 95 °C for 15 sec, annealing at 60 °C for 20 sec, and extension at 72 °C for 20 sec. GAPDH was used as the reference gene for normalization (Table 1).

### Statistical analysis

Normality of body weight and biochemical data was assessed using the Shapiro-Wilk normality test, and these data were found to be normally distributed. Statistical analysis for these data was performed using one-way analysis of variance (ANOVA) followed by Tukey’s *post hoc* test. Histopathological scores, as ordinal data, were analyzed using the Kruskal-Wallis test followed by Dunn’s *post hoc* test. Differences in mRNA expression levels between groups were analyzed using the 2((-ΔΔCT)) method. A p-value less than 0.05 was considered statistically significant. Data conforming to normal distribution are presented as mean ± standard deviation (SD), while non-normally distributed data are presented as median [interquartile range (IQR) – Q1, Q3]. All statistical analyses and calculations were performed using GraphPad Prism 8 and Microsoft Office 365 software.

## Results

The evaluation of body weights across the experimental groups revealed no statistically significant differences, indicating that ASTX administration did not notably influence body weight in the context of BPA-induced toxicity (*P*>0.05; Table 2).

### Effects of ASTX on oxidative stress and kidney damage biomarkers

**Figure 1:**
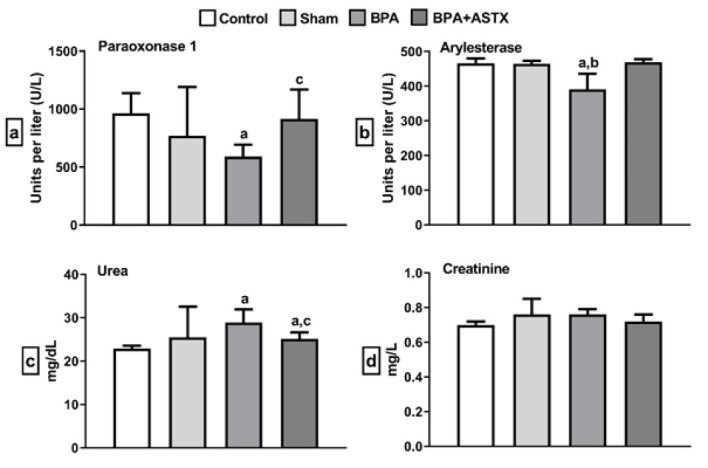
Parameters evaluated from blood serum using the enzyme-linked immunosorbent assay (ELISA) method

PON 1 and ARE results were significantly lower in the BPA groups compared to the control group (*P*<0.05). This suggests that BPA exposure may lead to an increase in oxidative stress, thereby contributing to oxidative damage. However, ASTX administration resulted in a statistically significant increase in PON 1 and ARE levels compared to the BPA-induced group (*P*<0.05). These findings suggest that ASTX may exert its protective effects by enhancing the activity of antioxidant enzymes to combat oxidative stress. Urea levels were significantly higher in the BPA groups compared to the control group (*P*<0.05). ASTX treatment restored the urea levels to those observed in the control group. No statistically significant differences were observed in creatinine levels (*P*>0.05) (Figure 1). The lack of change in creatinine levels may support the hypothesis that the differences in creatinine levels observed in previous studies are dose-dependent, related to the BPA concentration.

### Histopathologic staining

*Hematoxylin-eosin staining*

**Figure 2:**
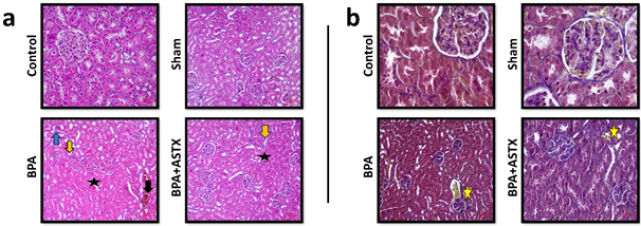
a. Pathological changes such as dilation (star), vascular congestion (black arrow), tubular epithelial desquamation (yellow arrow), and mononuclear cell infiltration (blue arrow) were observed in Wistar Albino rat kidney tissues stained with Hematoxylin and Eosin (H&E). b. Collagen fiber accumulation (yellow star) was observed in Masson's Trichrome-stained kidney sections

Light microscopic examination revealed that the glomeruli and renal tubules of the control group exhibited nearly normal structural organization in most kidneys. Kidney sections from the sham group also displayed typical histological renal architecture, similar to that observed in the control group. The BPA-treated group exhibited signs of vacuolization, congestion, tubular dilatation, desquamation, and infiltration. In contrast, the BPA+ASTX-treated group demonstrated a marked improvement in histological appearance compared to the BPA group. ASTX treatment was found to reduce the adverse effects induced by BPA on kidney tissue (Figure 2a, Table 3).

*Masson’s trichrome staining*

After Masson’s trichrome staining, no significant differences in fibrosis were observed in the kidney tissues of the control and sham groups. However, the BPA group exhibited an increase in fibrosis around the glomeruli and blood vessels. In the BPA+ASTX group, fibrosis was increased compared to the control and sham groups, but decreased when compared to the BPA group (Figure 2b).

### Immunohistochemical staining for bax and Bcl-2

**Figure 3:**
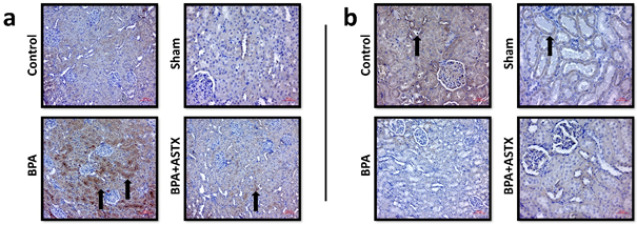
Effect of BPA exposure on apoptotic activity in Wistar Albino rat kidney tissue was evaluated through immunohistochemical expression of the pro-apoptotic protein Bax and the anti-apoptotic protein Bcl-2

When compared to all other groups, the BPA group exhibited significantly higher expression levels of Bax, particularly in the renal tubules. ASTX treatment was found to reduce Bax expression around the tubules when compared to the BPA group (Figure 3a, Table 3). Conversely, immunohistochemical staining for Bcl-2 showed a decrease in Bcl-2 expression in the BPA group compared to all other groups (Figure 3b, Table 3). Our findings on Bax and Bcl-2 emphasize the increase in apoptotic activity in kidney tissue due to BPA exposure. As a consequence of the elevated apoptotic activity, the damage observed in renal tubules and the increase in fibrosis are consistent with our histopathological findings.

### mRNA expression analysis of apoptosis and collagen-related genes

**Figure 4:**
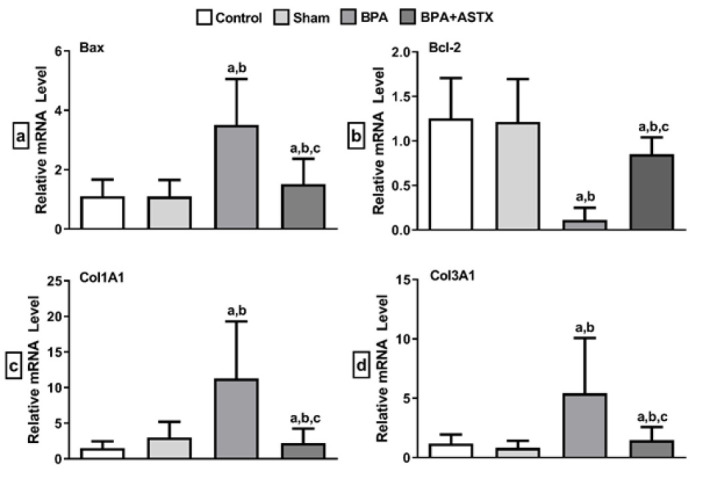
mRNA expression analysis a. Bax, b. Bcl-2, c. Col1A1, d. Col3A1

The effects of BPA and BPA+ASTX on the mRNA expression of apoptosis-related genes (Bax, Bcl-2) and collagen-related genes (Col1A1, Col3A1) were evaluated using RT-qPCR. Bax gene expression in the BPA group was significantly increased by 3.186-fold (*P*<0.05) compared to the control group and by 3.198-fold (*P*<0.05) compared to the sham group. In the BPA+ASTX group, Bax gene expression was reduced by 2.31-fold (*P*<0.05) compared to the BPA group (Figure 4a).

Bcl-2 gene expression in the BPA group was significantly reduced by 11.02-fold (*P*<0.05) compared to the control group and by 10.68-fold (*P*<0.05) compared to the sham group. When compared to the BPA group, Bcl-2 gene expression was significantly increased by 7.48-fold (*P*<0.05) in the BPA+ASTX group. No significant changes in Bcl-2 gene expression were observed when the BPA+ASTX group was compared separately to the control and sham groups (Figure 4b).

Col1A1 gene expression in the BPA group was significantly increased by 7.37-fold (*P*<0.05) compared to the control group and by 3.77-fold (*P*<0.05) compared to the sham group. In the BPA+ASTX group, Col1A1 mRNA expression was decreased by 5.09-fold (*P*<0.05) compared to the BPA group (Figure 4c). Col3A1 gene expression in the BPA-treated group was significantly increased by 4.58-fold (*P*<0.05) compared to the control group and by 6.63-fold (*P*<0.05) compared to the sham group. In contrast, Col3A1 mRNA expression was reduced by 3.7-fold (*P*= 0.05) in the BPA+ASTX group compared to the BPA group (Figure 4d).

## Discussion

BPA is among the most extensively investigated EDCs, with its adverse effects well-documented in various laboratory animal models and sentinel human populations (33). Recent studies have demonstrated that BPA and structurally related environmental contaminants exacerbate oxidative stress within cells by compromising the endogenous antioxidant defense mechanisms, thereby promoting the accumulation of ROS and the generation of deleterious metabolic byproducts (34). While the hepatotoxic effects of BPA have been extensively characterized, data regarding its nephrotoxic potential remain comparatively scarce. The present study aims to evaluate the therapeutic efficacy of ASTX in mitigating BPA-induced oxidative damage in renal tissue, with a particular focus on histopathological alterations and functional impairments in kidney physiology. 

Studies emphasize that ASTX exerts nephroprotective effects in various kidney pathologies through its antioxidant and anti-inflammatory properties. A key role in these effects is played by ASTX’s regulation of oxidative and inflammatory responses via the Nrf2/Keap1 or c-Src/Connexin 43 pathways. Additionally, ASTX’s nephroprotective effect is also demonstrated through a different mechanism by reducing mitochondrial ROS overproduction and inhibiting mitochondrial oxidative phosphorylation (35). Our study is based on the premise that BPA, a toxic substance, can cause oxidative damage, and consequently, oxidative injury may occur. It is designed around the hypothesis that ASTX may have therapeutic effects through the mechanisms described above.

Consistent with our findings, several researchers have reported that BPA exposure in rats and mice results in negligible differences in final body weight (36, 37). In contrast, other studies have demonstrated significant alterations in body weight, weight gain, and relative organ weights (38, 39). These inconsistencies in weight-related outcomes may be attributed to variations in dose, route of administration, exposure duration and timing, as well as differences in animal strains, sex, and dietary composition. Studies have indicated that elevated BPA levels are associated with a reduction in glomerular filtration rate, and increasing systemic BPA concentrations have even been proposed as potential biomarkers of renal injury (40). Therefore, investigations exploring the link between BPA exposure and kidney damage are crucial for the preservation of healthy renal function. PON1 and ARE are calcium-dependent antioxidant enzymes, localized in high-density lipoprotein (HDL), that function in esterase form and play a pivotal role in reducing oxidative stress in serum and tissues. These enzymes have been shown to hydrolyze hydrogen peroxide, the most commonly produced ROS, thereby preventing lipid peroxidation. Notably, their activities have been reported to decline in various pathological conditions (41). Ola-Davies and Olukole reported that oral administration of BPA at a dose of 10 mg/kg increased renal ROS levels while reducing the concentrations of endogenous antioxidants (42). Similarly, Kobayashi *et al*. observed that BPA exposure elevated the levels of plasma free radicals in rats (43). In line with these findings, Acaroz *et al*. demonstrated that BPA exposure exacerbated oxidative damage and decreased antioxidant enzyme levels (30). Consistent with the existing literature, our study also revealed that BPA exposure significantly reduced PON1 and ARE activities. However, supplementation with ASTX resulted in a marked increase in both PON1 and ARE capacities, a statistically significant finding.

Previous studies have reported that BPA exposure can lead to hyperglycemia, which may contribute to renal injury and dysfunction, ultimately resulting in significant increases in serum urea, creatinine, and uric acid levels (1). Suberg *et al*. and Saleh observed elevated serum urea and creatinine levels following oral administration of BPA, suggesting impaired renal filtration capacity (44, 45). In a separate study by Esplugas *et al*., mice exposed to a relatively low dose of BPA (25 mg/kg) exhibited signs of kidney damage, along with a significant increase in serum urea levels (46). However, no notable alterations were observed in creatinine concentrations or plasma clearance, indicating a dose- and biomarker-specific response to BPA-induced nephrotoxicity. Our findings are consistent with these studies, as BPA administration in our model also resulted in increased serum levels of renal function markers. Furthermore, ASTX supplementation at a dose of 20 mg/kg demonstrated a protective effect, which aligns with previous research indicating its efficacy in alleviating ROS-mediated oxidative stress and restoring the suppressed activity of endogenous antioxidant enzymes (47). In our findings, ASTX supplementation was shown to attenuate the BPA-induced elevation in serum urea levels, bringing them closer to those observed in the control group. This result was supported by statistical analysis. However, BPA exposure did not result in a statistically significant alteration in serum creatinine levels. This suggests that while urea may serve as a more sensitive early indicator of BPA-related renal impairment in this model, creatinine levels remained within a range that may not reflect subtle or early-stage nephrotoxicity.

Histopathological evidence from previous studies supports the nephrotoxic effects of BPA exposure. Dokmeci *et al*. (48) conducted H&E staining on renal sections of BPA-exposed rats and reported findings such as tubular epithelial desquamation, cytoplasmic vacuolization, and shrinkage. Similarly, Eid *et al*. (49) observed thickening of the glomerular basement membrane, increased cellular proliferation, and shedding of renal tubular microvilli in response to BPA exposure. Saleh *et al*. also reported a marked increase in collagen fiber deposition in renal tissues of BPA-treated animals compared to controls, suggesting progressive fibrotic changes (50). In contrast, studies investigating the protective effects of ASTX have reported its beneficial influence on renal histopathology under various pathological conditions. In a model of alloxan-induced diabetes, ASTX administration significantly improved histological damage such as glomerular and tubular degeneration, an effect attributed to its potent antioxidant properties (51). Erbaş *et al*. demonstrated that ASTX preserved normal renal histology in the context of lithium-induced toxicity, which was evidenced by reduced glomerular and tubular degeneration as well as diminished edema (22). These findings are in line with broader evidence suggesting that ASTX exerts protective effects against oxidative stress-induced cellular and tissue damage in both *in vitro* and *in vivo* models. Furthermore, ASTX has been reported to protect proximal tubular epithelial cells and diabetic nephropathy models from oxidative stress, inflammation, and apoptosis induced by high glucose levels (47).

In our study, ASTX administration was found to exert a protective effect on renal tissue by mitigating the histological alterations induced by BPA exposure. Based on our findings, it is believed that the observed renal damage may be primarily attributed to oxidative stress triggered by BPA, and that ASTX plays a significant role in attenuating this damage through its potent antioxidant activity. Consistent with our Masson’s trichrome staining results, the BPA+ASTX group exhibited reduced fibrotic tissue deposition compared to the BPA group, which demonstrated a markedly increased degree of fibrosis. This histological improvement in the ASTX-treated group suggests a potential antifibrotic role of ASTX in the context of BPA-induced renal injury. Furthermore, molecular analysis revealed a significant up-regulation of fibrosis-associated genes Col1A1 and Col3A1 in the BPA group, whereas their expression was notably down-regulated in the BPA+ASTX group. These findings support the hypothesis that ASTX not only modulates oxidative stress but also influences the transcriptional regulation of key fibrogenic pathways, thereby contributing to the preservation of renal structural integrity.

BPA exposure has been shown to down-regulate the expression of PI3K (phosphatidylinositol 3-kinase) and AKT (protein kinase B), while activating the Bcl-2/Bax–Caspase-9–Caspase-3 signaling cascade, ultimately leading to apoptosis and necrosis of renal cells (52). Tang *et al*. reported that the increase in apoptotic activity due to BPA exposure is accompanied by a disruption in mitochondrial membrane potential (53). Ma *et al*. reported that exposure to toxic substances activates the p53 pathway, leading to increased apoptotic activity in kidney tissue. It is thought that the increase in apoptotic activity highlighted by our study’s results may have occurred through similar mechanisms (54). Bosch-Panadero *et al*. also reported increased apoptotic activity in proximal tubular cells following BPA exposure (55). In line with these findings, Amin *et al*. demonstrated that oral administration of BPA at a dose of 50 mg/kg in male albino rats significantly up-regulated the expression of pro-apoptotic Bax protein and profibrotic TGF-β1, along with histopathological evidence of apoptosis and fibrosis (56). Supporting our results, previous studies have indicated that ASTX exerts cytoprotective effects on renal cells by preserving mitochondrial function and maintaining mitochondria in a more reduced state under oxidative stress conditions (22). Consistent with the study by Guo *et al*., which demonstrated that ASTX significantly reduced the number of apoptotic renal cells, an effect attributed to its inhibition of tubular apoptosis, our findings similarly showed that BPA exposure increased Bax expression and decreased Bcl-2 expression in renal tissue, as verified by both immunohistochemical staining and PCR analysis (47). Significantly, ASTX administration reversed these molecular alterations, resulting in down-regulation of Bax and up-regulation of Bcl-2 expression. These findings suggest that ASTX may reduce BPA-induced renal apoptosis by regulating the intrinsic apoptotic pathway, potentially through stabilization of mitochondrial integrity and inhibition of pro-apoptotic signaling. Consequently, the reduction of increased apoptotic activity caused by BPA exposure via ASTX treatment may have occurred through suppression of mitochondrial oxidative phosphorylation and mitochondrial oxidative overproduction, or inhibition of the p53 signaling pathway.

Studies have emphasized a strong association between BPA exposure and kidney pathologies in humans (57). Furthermore, individuals with chronic kidney disease (CKD) have been reported to be potentially at higher risk for adverse effects resulting from BPA exposure (58). The reported impacts of BPA exposure in humans align with our findings, thereby reinforcing the conclusion that BPA induces nephrotoxicity. Consequently, the development of protective, preventive, and therapeutic strategies against the detrimental effects of BPA exposure is of paramount importance in the current context.

BPA exposure emerges as a factor affecting kidney health in individuals. It is crucial to conduct qualified, long-term, and methodologically diverse studies for clinical practice and policy-making. Although ASTX shows promise in terms of safety and biological efficacy, particularly regarding kidney health, larger and more robust studies are needed to confirm its clinical benefits. In conclusion, while there is robust preclinical evidence supporting the protective effects of the BPA–ASTX interaction, confirming this effect through feasible, randomized clinical trials from a human health perspective will be a critical step in the future.

**Table 1:** Primer sequences of apoptosis and collagen-related target and reference genes used in RT-qPCR analysis

Gene	Forward primer (5′- >3′)	Reverse primer (5′- >3′)
*BAX*	GATGGCCTCCTCCTTTCCTACTTC	CTTCTTCCAGATGGTGAGTGAG
*BCL2*	GGAGGATTGTGGCCTTCTTCTTT	GTCATCCACAGAGCGATGTT
*COL1A1*	CCAATGGTGCTCCTGGTATT	GTTCACCACTGTTGCCTTTG
*COL3A1*	GTGTGATGATGATGAGCCACTAGAC	TGACACAGGAGCAGGAGGTGTAGAA
*GAPDH*	GCATTGCAGAGGATGGTAGAGAG	GCGGGAGAAGAAAGTCATGATTAG

**Table 2:** Initial and final body weights of rats

	Control	Sham	BPA	BPA+ASTX	*p*
First	221.14±16.94 g	217.78±12.90 g	238.13±18.88 g	234.33±22.37 g	0.3360
Last	240.80±24.85 g	231.10±24.65 g	253.00±26.61 g	237.13±21.32 g	0.3136

Data were given as mean ± standard deviation (SD). To compare the experimental groups, a one-way analysis of variance (ANOVA), a parametric test for independent groups, was applied. *Post hoc *comparisons between pairs of groups were performed using Tukey’s HSD test. *P*-values refer to the ANOVA.

a. Serum Paraoxonase 1 (PON 1) levels, b. Serum Arylesterase (ARE) levels, c. Serum Urea levels, d. Serum Creatinine levels

Data are presented as mean ± standard deviation (SD). To compare the experimental groups, one-way analysis of variance (ANOVA), a parametric test for independent groups, was applied. *Post hoc* comparisons between pairs of groups were performed using Tukey’s HSD test. *P*-values refer to the ANOVA.

a: Statistically significant compared to the Control group (*P*<0.05).

b: Statistically significant compared to the Sham group (*P*<0.05).

c: Statistically significant compared to the BPA group (*P*<0.05).

Accordingly, an increase in fibrosis was seen in the BPA group, while a decrease was noted in the BPA+ASTX group. BPA+ASTX: Bisphenol A + Astaxanthin

a. Immunohistochemically stained kidney sections indicated by the black arrow show a significant increase in Bax expression following BPA exposure, attributable to elevated oxidative stress and reactive oxygen species (ROS), which disrupt mitochondrial membrane potential. This disruption triggers the mitochondrial apoptotic pathway, leading to activation of the intracellular caspase cascade. Administration of astaxanthin (ASTX), with its potent free radical scavenging activity, effectively reduced ROS levels, preserved mitochondrial membrane stability, and consequently significantly decreased Bax expression. b. In the same sections, Bcl-2 expression indicated by the black arrow was diminished due to BPA-induced suppression of anti-apoptotic signaling pathways. ASTX treatment mitigated oxidative stress, supported mitochondrial function, and increased Bcl-2 expression, thereby enhancing cellular resistance to apoptosis. BPA: Bisphenol A; ASTX: Astaxanthin

Data are presented as mean±standard deviation (SD)

Therefore, a one-way analysis of variance (ANOVA), a parametric test for independent groups, was applied to compare the experimental groups. Post hoc comparisons between pairs of groups were performed using Tukey’s HSD test. *P*-values refer to the ANOVA.

a: Statistically significant compared to the Control group (*P*<0.05)

b: Statistically significant compared to the Sham group (*P*<0.05)

c: Statistically significant compared to the BPA group (*P*<0.05)

**Table 3:** Histopathological scores obtained from hematoxylin & eosin (H&E) staining images and scores obtained from Bax and Bcl-2 expression from immunohistochemical staining images of Wistar albino rats

	Histopathological score Median [Q1-Q3]	Bax score Median [Q1-Q3]	Bcl-2 score Median [Q1-Q3]
Control	1.5 [1.16-2.0]	0.50 [0.33-0.50]	2.5 [2.25-2.66]
Sham	1.58 [1.25-2.0]	0.50 [0.37-0.66]	2.5 [2.37-2.62]
BPA	13.33 [13.0-13.46](a,b)	2.83 [2.66-2.96](a,b)	0.42 [0.33-0.50](a,b)
BPA+ASTX	4.42 [3.37-4.50]	1.50 [1.33-1.50]^a^	1.92 [1.83-2.12]
	*P*<0.0001	*P*<0.0001	*P*<0.0001

Data are presented as median [interquartile range (IQR)]. Statistical analysis was performed using the Kruskal–Wallis test followed by Dunn’s* post hoc *test

*P*-values refer to the Kruskal–Wallis test. a: Statistically significant compared to the Control group (*P*<0.05). b: Statistically significant compared to the Sham group (*P*<0.05).

## Conclusion

This study was conducted on adult male rats and did not address potential differences related to age and sex. Moreover, it was carried out with a limited sample size. The dose–response relationships of both BPA exposure and ASTX supplementation need to be further explored in future studies. Additionally, the findings of this study are based solely on kidney tissue analysis. Investigations involving other tissues and different species may be considered in subsequent research. Nonetheless, our results suggest that ASTX supplementation may alleviate BPA-induced histopathological alterations and protect kidney tissue. These findings indicate that ASTX holds promise as a potential therapeutic or protective agent against BPA-induced nephrotoxicity. However, further studies are needed to elucidate the precise mechanisms underlying its protective effects.

## Data Availability

The raw data supporting the conclusions of this article will be made available by the authors upon request.

## Declaration

We acknowledge the use of GPT-4o to suggest corrections to English grammar.
